# Characterization of sclerostin’s response within white adipose tissue to an obesogenic diet at rest and in response to acute exercise in male mice

**DOI:** 10.3389/fphys.2022.1061715

**Published:** 2023-01-04

**Authors:** Nigel Kurgan, Bradley Baranowski, Joshua Stoikos, Adam J. MacNeil, Val A. Fajardo, Rebecca E. K. MacPherson, Panagiota Klentrou

**Affiliations:** ^1^ Department of Kinesiology, Brock University, St. Catharines, ON, Canada; ^2^ Centre for Bone and Muscle Health, Brock University, St. Catharines, ON, Canada; ^3^ Department of Health Sciences, Brock University, St. Catharines, ON, Canada

**Keywords:** sclerostin, Wnt signalling, GSK3, adipose tissue, exercise

## Abstract

**Introduction:** It is well established that sclerostin antagonizes the anabolic Wnt signalling pathway in bone, however, its physiological role in other tissues remains less clear. This study examined the effect of a high-fat diet (HFD) on sclerostin content and downstream markers of the Wnt signaling pathway (GSK3β and β-catenin) within subcutaneous inguinal white adipose tissue (iWAT), and visceral epididymal WAT (eWAT) depots at rest and in response to acute aerobic exercise.

**Methods:** Male C57BL/6 mice (*n* = 40, 18 weeks of age) underwent 10 weeks of either a low-fat diet (LFD) or HFD. Within each diet group, mice were assigned to either remain sedentary (SED) or perform 2 h of endurance treadmill exercise at 15 m min^−1^ with 5° incline (EX), creating four groups: LFD + SED (*N* = 10), LFD + EX (*N* = 10), HFD + SED (*N* = 10), and HFD + EX (*N* = 10). Serum and WAT depots were collected 2 h post-exercise.

**Results**: Serum sclerostin showed a diet-by-exercise interaction, reflecting HFD + EX mice having higher concentration than HFD + SED (+31%, *p* = 0.03), and LFD mice being unresponsive to exercise. iWAT sclerostin content decreased post-exercise in both 28 kDa (−31%, *p* = 0.04) and 30 kDa bands (−36%, main effect for exercise, *p* = 0.02). iWAT β-catenin (+44%, *p* = 0.03) and GSK3β content were higher in HFD mice compared to LFD (+128%, main effect for diet, *p* = 0.005). Monomeric sclerostin content was abolished in eWAT of HFD mice (−96%, main effect for diet, *p* < 0.0001), was only detectable as a 30 kDa band in LFD mice and was unresponsive to exercise. β-catenin and GSK3β were both unresponsive to diet and exercise within eWAT.

**Conclusion**: These results characterized sclerostin’s content to WAT depots in response to acute exercise, which appears to be specific to a reduction in iWAT and identified a differential regulation of sclerostin’s form/post-translational modifications depending on diet and WAT depot.

## Introduction

White adipocytes are a major storage site for lipids and play a key role in substrate mobilization (e.g., fatty acids) to tissues (e.g., skeletal muscle) during periods of high energy expenditure (Lundsgaard et al., 2018). When energy intake is in excess of expenditure and is sustained, adipose tissue expansion occurs through the hypertrophy and hyperplasia of adipocytes (Spalding et al., 2008; Arner et al., 2013; Wang et al., 2013). Despite a growing understanding of the development of adipose tissue depots, the mechanisms and signals regulating these responses according to changes in energy needs (e.g., obesogenic diet or exercise) are not fully understood.

Sclerostin is a glycoprotein, that is, mainly associated with the regulation of bone metabolism. In the bone microenvironment, osteocytes secrete sclerostin in response to mechanical unloading, which inhibits Wnt/β-catenin signaling within the bone-forming cells, the osteoblasts. Wnt signaling is initiated when Wnt ligands bind to the Frizzled (Fz) receptor and its co-receptors low-density lipoprotein receptor-related protein 5 and 6 (Zeng et al., 2005). After activation, the β-catenin destruction complex, composed of Axin, adenomatous polyposis coli (APC), casein kinase 1, and glycogen synthase kinase 3 (GSK3), becomes dissociated. This enables the accumulation of β-catenin due to the inhibition of GSK3 mediated β-catenin phosphorylation and subsequent proteasomal degradation (Taelman et al., 2010). Inhibiting Wnt/β-catenin signaling by sclerostin leads to the inhibition of bone formation and the subsequent upregulation of the activation of the bone-resorbing cells, the osteoclasts (Baron et al., 2006). While studies have shown lower expression of sclerostin by the liver, kidney, both the vascular smooth muscle (Zhu et al., 2011) and skeletal muscle (Magarò et al., 2021), testis, pyloric sphincter, carotid arteries, and the cerebellum (Collette et al., 2013), the majority of circulating sclerostin comes from bone (Kim et al., 2019; Kim et al., 2021). While most studies show adipose tissue does not express sclerostin (Kim et al., 2017), there is some evidence that there are detectable transcript levels within adipose tissue depots in adult mice (Gene ID: 74499 in NIH GEO Profiles). However, sclerostin knockout mice or mice with peripheral inhibition of sclerostin by a neutralizing antibody present with increased bone mass, as well as reduced peripheral fat pad mass, increased insulin sensitivity, and fatty acid oxidation (Kim et al., 2017; Kim et al., 2021). These findings suggest an endocrine role of bone derived sclerostin in regulating adipose tissue mass and metabolism. Sclerostin’s endocrine role in adipose tissue is likely mediated in part by inhibiting Wnt signaling, a pathway known to inhibit adipogenesis (Cawthorn et al., 2012). Indeed, when sclerostin content is increased both *in vivo* (Fulzele et al., 2017; Kim et al., 2017; Fairfield et al., 2018) and *in vitro* (Ukita et al., 2016), Wnt signaling is inhibited, resulting in increased adipogenesis and adipocyte cell size.

Evidence for a physiological role of sclerostin in regulating fat mass comes from assessment of humans with higher adiposity (Amrein et al., 2012; Urano et al., 2012) and mice with increased adiposity induced by a HFD, which both have higher circulating sclerostin compared to their normal weight controls (Baek et al., 2014; Kim et al., 2017). Exercise differentially influences circulating sclerostin levels; specifically, acute exercise induces a transient increase (Kouvelioti et al., 2019), while resting levels decrease with long term exercise training (Ardawi et al., 2012). Additionally, adolescent females appear to be protected from this increase while obese adolescent females are not (Kurgan et al., 2020), providing evidence for adiposity dependent differential mobilization of sclerostin in the circulation following acute exercise. This differential systemic mobilization of sclerostin may be related to its impact on insulin sensitivity and glucose metabolism, which has been shown in humans (Urano et al., 2012; Daniele et al., 2015) and *in vivo* murine models (Kim et al., 2017; Kim et al., 2019; Kim et al., 2021). Taken together, these data suggest sclerostin’s endocrine function may regulate adipose tissue mass and metabolism in response to excess energy intake (e.g., HFD) and energy expenditure (e.g., exercise). Examining the content of sclerostin to different WAT depots in response to acute exercise or to chronic changes in energy availability (e.g., HFD) will provide physiological context to sclerostin’s endocrine function. The purpose of this study was to examine subcutaneous and visceral WAT sclerostin content and Wnt/β-catenin signaling in response to a HFD and determine whether the subsequent metabolic adaptations (reduced insulin sensitivity/dysregulated glucose homeostasis) influence the post-exercise response in WAT sclerostin content.

## Materials and methods

### Animals

Experimental protocols are in compliance with the Canadian Council on Animal Care and were approved by the Brock University Animal Care Committee (File #17-06-02). This study utilized one cohort of 40 male C57BL/6 mice (18 weeks of age; 31.9 ± 2.5 g) ordered from The Jackson Laboratory (Bar Harbor, Main, United States) for a larger study originally designed to examine brain signaling response to acute exercise (Baranowski et al., 2021). Upon arrival, the mice acclimatized for 3 days in the Brock University Comparative Biosciences Facility (St. Catharines, Ontario, Canada). During acclimatization, mice were fed standard chow (2014 Teklad global 14% protein rodent maintenance diet, Harlan Tekland, Mississauga, Ontario, Canada). All mice were housed in fours, were kept on a 12 h light/12 h dark cycle and had *ad libitum* access to food and water through the entirety of the study.

### Experimental design

All materials used are listed in Supplementary Table S1. As previously described (Baranowski et al., 2021), mice were randomized into either a HFD (*n* = 20, 60% kcal fat, cat#D12492, Research Diets Inc. New Brunswick, New Jersey, United States) or low-fat diet (LFD) group (*n* = 20, 10% kcal fat, cat#D12450B, Research Diets Inc. New Brunswick, New Jersey, United States). Baseline measurement of body mass of all mice (*n* = 40) was conducted immediately after acclimatization and the mice remained on their respective diets for a 10-week period, with body mass measurements taken weekly. Following the 10-week dietary intervention, LFD and HFD mice were further randomized into either the sedentary or the acute exercise group, creating four groups: LFD + Sedentary (*n* = 10), LFD + Exercise (*n* = 10), HFD + Sedentary (*n* = 10), or HFD + Exercise (*n* = 10). The sedentary groups did not exercise, while the exercise groups performed a single bout of treadmill running for 2 h at 15 m min^−1^ on a 5% incline as previously described (MacPherson et al., 2015). Following a 2 h recovery period, the mice were euthanized, and serum, as well as the inguinal white adipose tissue (iWAT) and epididymal white adipose tissue (eWAT) depots, were collected. Necropsy tissue masses were also measured prior snap freezing in liquid nitrogen and stored at −80°C for future analysis.

### Glucose and insulin tolerance testing

Data from the glucose (GTT) and insulin tolerance testing (ITT) from a larger cohort of animals is described and reported elsewhere (Baranowski et al., 2021). Briefly, intraperitoneal GTT were performed on fasted (6 h), non-anesthetized mice during the 9th week of the dietary intervention. Tail vein was sampled for blood glucose using an automated glucometer at baseline and at 15, 30, 45, 60, 90, and 120 min following an intraperitoneal injection of glucose (2 g kg body mass^−1^) and area under the curve (AUC) of the glucose response over time is reported in Table 1 (Baranowski et al., 2021).

**TABLE 1:** Differences between the low fat and high fat diet groups in body mass, tissue necropsy, and insulin sensitivity measures following 8 weeks of dietary intervention.

	Low fat diet	High fat diet	*p*-value
Body Mass (g)	32.2 ± 3.2	48.4 ± 5.2	<0.0001
iWAT Mass (g)	0.4 ± 0.4	1.9 ± 0.5	<0.0001
eWAT Mass (g)	0.8 ± 0.4	1.6 ± 0.3	0.0003
Liver Mass (g)	1.1 ± 0.2	2.4 ± 0.6	<0.0001
ITT AUC (mmol·L^-1^·min^-1^)	520.4 ± 80.3	955.6 ± 84.6	<0.0001
GTT AUC (mmol·L^-1^·min^-1^)	1299.1 ± 159.6	2057.3 ± 286.5	<0.0001

Data are means ± SD., Independent sample *t*-tests were used to examine group differences. iWAT, inguinal white adipose tissue; eWAT, epididymal white adipose tissue; ITT, insulin tolerance test; AUC, area under the curve; GTT, glucose tolerance test. Data is a cohort of previously published data (Baranowski et al., 2021).

Intraperitoneal ITT were performed on non-anesthetized mice following 48 h of recovery from the GTT. Tail vein was sample for blood glucose using an automated glucometer at baseline and at 15, 30, 45, 60, 90, and 120 min following an intraperitoneal injection of insulin (0.75 U kg body mass^−1^) and (AUC) of the glucose response over time is reported in Table 1 (Baranowski et al., 2021).

### Blood sampling and ELISA

At end point, blood samples were taken by cardiac puncture with 1 ml insulin syringes (cat#01ST; ELIMEDICAL, Markham, Ontario, Canada) and 25G needles (cat#26403; EXCELINC International,Co., California, United States). Blood samples were left on ice to clot for 1 h before being centrifuged for 15 min at x1500 g. Samples were aliquoted and stored at −80°C until analysis. Serum sclerostin was analysed in duplicate using one Quantikine enzyme-linked immunoassay kits (cat#MSST00; R&D Systems, Minneapolis, Minnesota, United States) according to manufacturer’s instructions and had an intra assay coefficient of variation of 4.8%.

### Adipose tissue homogenization

iWAT and eWAT depots were tandemly homogenized (FastPrep^®^, MP Biomedicals, Santa Ana, CA) on the same day in 3x volume per mg mass of tissue in NP40 Cell Lysis Buffer (cat# FNN0021, Life Technologies, Carlsbad, California, United States) supplemented with 3x the recommended volume of phenylmethylsulfonyl fluoride (PMSF) (3 mM) and 100x protease inhibitor (PI) cocktail (cat#7626 and cat#P8340, respectively, Sigma-Aldrich, St. Louis, Missouri, United States). Following homogenization, samples were placed on ice for 30 min then centrifuged at 4°C for 5 min at x5,000 g. The infranatant was then collected and protein concentration was determined using a Bicinchoninic acid assay (cat#B9643; Sigma-Aldrich, St. Louis, Missouri, United States; copper (II) sulfate pentahydrate, cat#BDH9312, VWR, Radnor, Pennsylvania, United States). The samples were prepared to contain equal concentrations (1 μg μl^−1^) of protein in 4x Laemmli buffer (diluted to 1x; cat#1610747, Bio-Rad, Hercules, California, United States) and placed in a heating block at 100°C for 10 min, allowed to cool, and then stored at −80°C for future analysis.

### Immunoblotting

20 µg of protein were loaded and resolved on 10% TGX fast cast gels (cat# 1610173, Bio-Rad, Hercules, California, United States) for 30 min at 250 V. Proteins were then semi-dry transferred onto a polyvinylidene difluoride membrane at 1.3 A and 25 V for 7 min (Trans-Blot^®^ Turbo™ Transfer System, Bio-Rad, Hercules, California, United States). Membranes were blocked in Tris buffered saline/0.1% Tween 20 (TBST) with 5% non-fat powdered milk for 1 h at room temperature. Primary antibody (1:500–2000 ratio dilution in 5% milk) was then applied and left to incubate on a shaker at 4°C for ∼16 h. Membranes were then washed with TBST 3 × 5 min and then incubated with the corresponding secondary antibody conjugated with horseradish peroxidase (anti rabbit, cat#HAF008, and anti-goat, cat#CAF109, R&D Systems, Minneapolis, Minnesota, United States—1:2000 dilution in 5% milk) for 1 h at room temperature. Signals were detected using either Clarity™ Western chemiluminescent substrate (cat#170-5061, Bio-Rad, Hercules, California, United States), or SuperSignal™ West Femto maximum sensitivity chemiluminescent substrate (cat#34095, ThermoFisher Scientific, Waltham, Massachusetts, United States) and were imaged using a Bio-Rad ChemiDoc™ Imaging System (Hercules, California, United States). Densitometry analysis was done using ImageLab Software (Bio-Rad, Hercules, California, United States) and compared to sedentary LFD mice protein content (e.g., 100%). Antibodies against total GSK3β (cat#12456S), serine 9 phospho-GSK3β (cat#9336S), total β-catenin (cat#8480S) were purchased from Cell Signalling (Danvers, Massachusetts, United States). Vinculin (cat#ab129002) was used as loading control and purchased from Abcam (Toronto, Ontario, Canada). Sclerostin antibody was purchased from R&D Systems, Inc. (cat#MAB1406, R&D Systems, Minneapolis, Minnesota, United States), and was confirmed by immunoblotting femur homogenates and recombinant sclerostin (cat# 1406, R&D Systems, Minneapolis, Minnesota, United States) (Supplementary Figure S1).

### Statistics

Baseline comparisons between diet groups were done using independent *t-*tests. Comparisons of all proteins responses to diet and exercise were done with a 2-way ANOVA (diet by exercise groups) and significant interactions were followed up with Tukey *post hoc* analysis using GraphPad Prism 9. Data are presented as means ± standard deviation (SD) with significance assumed at an α = 0.05.

## Results

### Diet induced changes to body mass and insulin sensitivity

Table 1 presents the differences between the LFD and HFD groups in body mass, tissue necropsy, and insulin sensitivity for the 40 mice included in the present study. It should be noted that since this was a subsample of the original cohort (*N* = 72) from the larger study (Baranowski et al., 2021), these variables were re-analyzed for the purpose of the present study. Mice on HFD had higher body mass (mean difference = +16.2 g or +1.5-fold, *p* < 0.0001), necropsy iWAT mass (mean difference = +1.5 g or +4.8-fold, *p* < 0.0001), necropsy eWAT mass (mean difference = +0.8 g or +2-fold, *p* = 0.0003), and necropsy liver mass (mean difference = +1.3 g or +2.2-fold, *p* < 0.0001) compared to mice on a LFD (Table 1). Mice on HFD also had higher area under the curves (AUC) of blood glucose in response to ITT (mean difference = +435.2 mmol L^−1^ min^−1^ or +1.8-fold, *p* < 0.0001) and GTT (mean difference = +758.2 mmol L^−1^ min^−1^ or +1.6-fold, *p* < 0.0001) compared to a LFD. These results provide evidence that our HFD resulted in fat mass expansion and perturbed insulin sensitivity\glucose metabolism.

### Circulating sclerostin increases acutely following exercise only in high-fat diet mice

**FIGURE 1:**
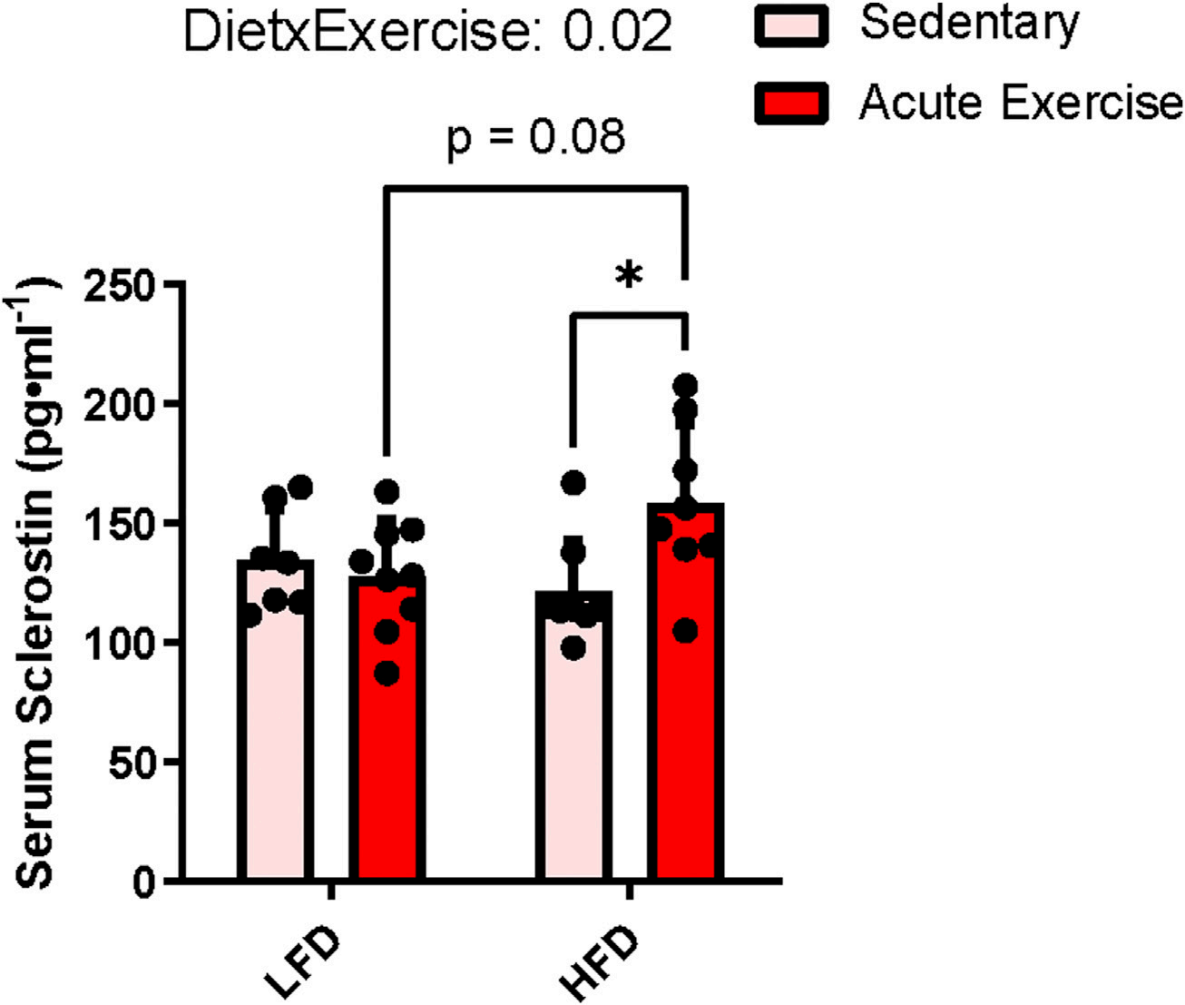
Serum sclerostin response to HFD and acute exercise. Light grey bars represent sedentary mice and dark grey bars represent mice sampled 2 h following 2 h of acute endurance exercise and are grouped by diet; LFD = low fat diet; HFD = high fat diet. Data are presented as means ± SD. A two-way factorial ANOVA was used to examine main effects for diet and exercise as well their interaction. *Tukey* correction was used for multiple pairwise comparisons.

Circulating sclerostin content showed no main effects for exercise (*p* = 0.1) or diet (*p* = 0.3), but there was a diet by exercise interaction (*p* = 0.02), indicating that the sclerostin response to acute exercise was different between diets. Pairwise comparisons identified that HE mice had higher serum sclerostin compared to HS (mean difference = +36.9 pg ml^−1^, *p* = 0.03) and trended to be significantly higher than LE mice (mean difference = +30.3 pg ml^−1^, *p* = 0.08), while there were no differences between LS and LE groups (Figure 1). These findings reflect similar results observed in humans with obesity compared to those with normal weight (Kurgan et al., 2020).

### Obesogenic diet increases GSK3β and β-catenin content and acute exercise reduces sclerostin content within inguinal white adipose tissue

When immunoblotting for sclerostin within iWAT, a distinct banding pattern occurred at 28 and 30 kDa, which represents glycosylation at N-linked sites (N175) (Krause et al., 2010; Holdsworth et al., 2012; Kim et al., 2020). There was also a band at ∼56 kDa, that we believe to be a sclerostin dimer (Hernandez et al., 2014). This band was haloing when the monomer was detectable, which could indicate a saturated signal and thus a higher abundance than the monomer (Supplementary Figure S3). Nevertheless, the monomeric form of sclerostin has been shown to be functional and interact specifically with the Wnt coreceptor (LRP6) inhibiting its action (Kim et al., 2020). Thus, we report here analysis only on the monomer of sclerostin, which was accomplished by analyzing membranes that had been cut below 56 kDa to only detect monomeric sclerostin without the haloing 56 kDa band (e.g., Supplementary Figure S2).

**FIGURE 2:**
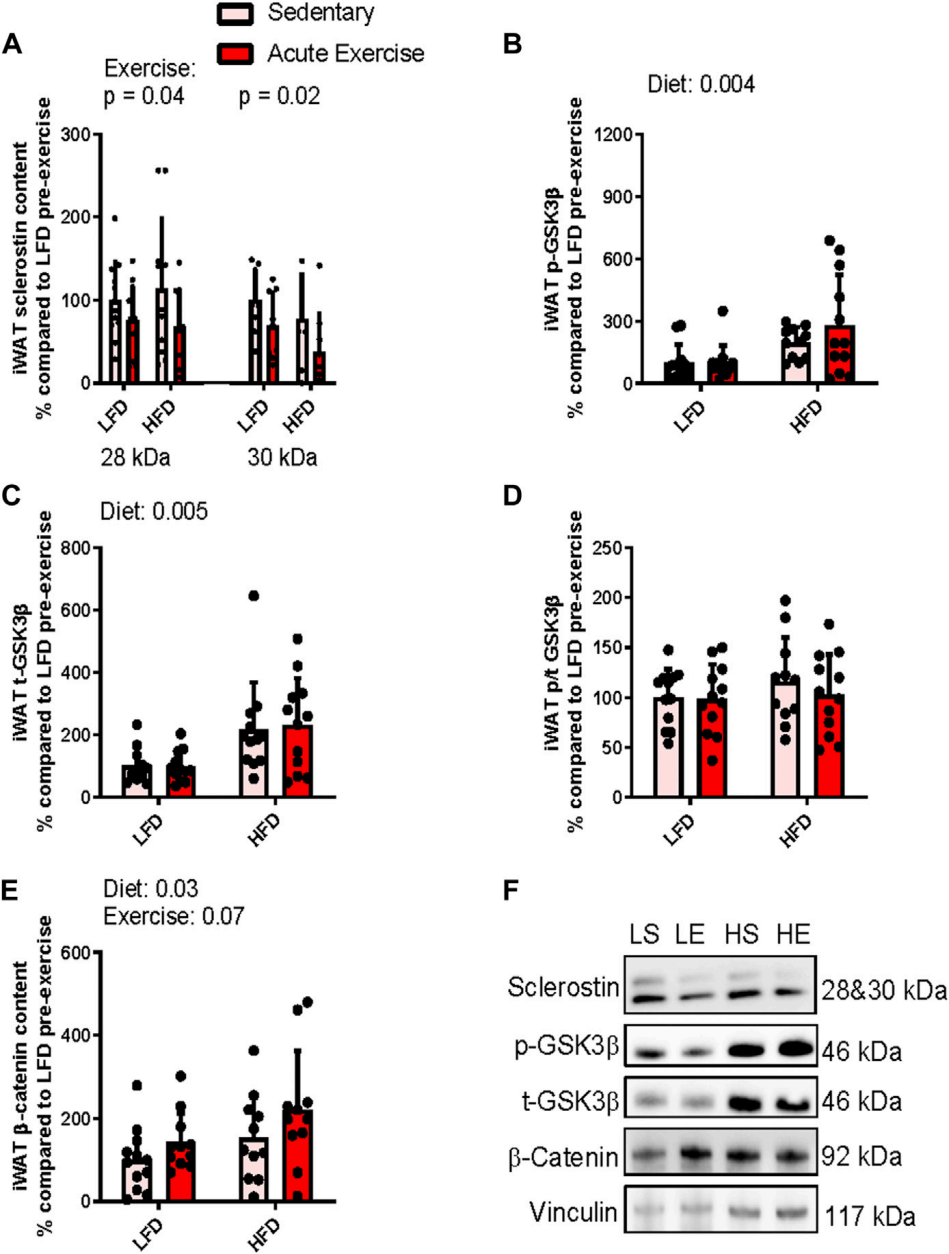
Response of iWAT sclerostin content **(A)**, serine9 phosphorylation status **(B,D)** both total GSK3b content **(C)**, and total b-catenin content **(E)** to HFD and acute exercise, as well as representative immunoblots **(F)**. Light grey bars represent sedentary mice and dark grey bars represent mice sampled 2 h following 2 h of acute endurance exercise and are grouped by diet; LFD = low fat diet; HFD = high fat diet. Proteins are corrected for by the house keeping protein vinculin and are all relative to LFD sedentary mice, data are presented as means ± SD and main effects are presented on graphs only if they are either close to or are significant. A two-way factorial ANOVA was used to examine main effects for diet and exercise as well their interaction. *Tukey* correction was used for multiple pairwise comparisons.

Examination of iWAT sclerostin’s 28 kDa band showed a main effect for exercise indicating that exercise, irrespective of diet, led to a ∼35% reduction (*p =* 0.04) in sclerostin content (Figure 2A). We did not observe a main effect for diet (difference in predicted means of LFD and HFD = +3.0%, *p* = 0.9) nor did we detect an interaction between exercise and diet (*p* = 0.5) (Figure 2A). Similarly, we detected a main effect of exercise for sclerostin’s 30 kDa band indicating a 35% reduction in response to exercise (*p* = 0.02, Figure 2A). Again, we did not detect a main effect for diet (difference in predicted means of HFD and LFD = +27.4%, *p* = 0.08) and no interaction between diet and exercise (*p* = 0.8) (Figure 2A).

To examine sclerostin’s downstream effect on Wnt signaling, we assessed Wnt mediated inactivation phosphorylation of GSK3. For serine 9 phosphorylation of GSK3, we found a ∼174% (*p* = 0.004) increase in response to the diet, irrespective of exercise, indicating that the HFD increased GSK3 phosphorylation (Figure 2B). There was no main effect for exercise (difference in predicted means from pre-to post-exercise = +7.1%; *p* = 0.9) and no interaction (*p* = 0.9) for serine9 phosphorylation of GSK3β within iWAT (Figure 2B). Likewise, there was a main effect for diet when we examined total GSK3β, suggesting that its content was increased ∼142% (*p =* 0.0005) in response to the HFD (Figure 2B). However, again we did not detect a main effect for exercise (difference in predicted means from pre-to post-exercise = +7.3%, *p* = 0.8) and no interaction (*p* = 0.7) for total GSK3β content within iWAT (Figure 2C). Together, these responses resulted in no main effects for either exercise (difference in predicted means from pre-to post-exercise = −8.1%, *p* = 0.4) or diet (difference in predicted means of LFD and HFD = +10.9%, *p* = 0.4) and interaction (*p* = 0.5) for serine9 phosphorylation/total GSK3β ratio within iWAT (Figure 2D).

Next, to see if there were any downstream activation of Wnt signaling, we examined accumulation of β-catenin. There was a main effect for diet, indicating that irrespective of exercise status, the HFD increased β-catenin by approximately 66% (*p* = 0.03, Figure 2E). There was no main effect for exercise (difference in predicted means from pre-to post-exercise = +55.4%, *p* = 0.07) and no interaction (*p* = 0.7) for total β-catenin content within iWAT (Figure 2E). Figure 2F presents representative immunoblots for Wnt signaling related proteins in iWAT from each group. se.

### Obesogenic diet abolishes sclerostin content within epididymal white adipose tissue and results in a differential response to acute exercise in inhibitory GSK3β serine9 phosphorylation status

**FIGURE 3:**
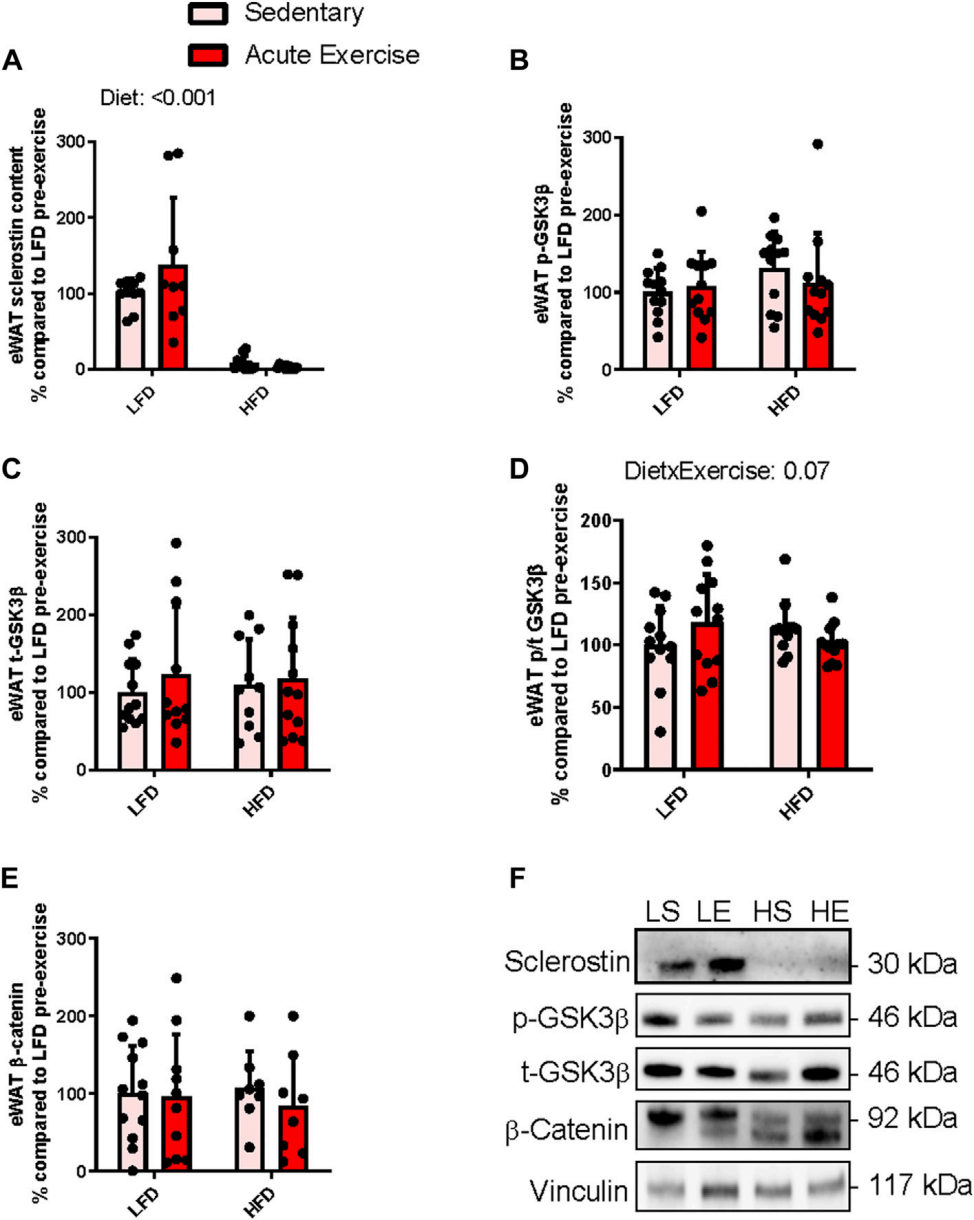
Response of eWAT sclerostin content **(A)**, serine9 phosphorylation status **(B,D)** both total GSK3b content **(C)**, and total b-catenin content **(E)** to HFD and acute exercise, as well as representative immunoblots **(F)**. Light grey bars represent sedentary mice and dark grey bars represent mice sampled 2 h following 2 h of acute endurance exercise and are grouped by diet; LFD = low fat diet; HFD = high fat diet. Proteins are corrected for by the house keeping protein vinculin and are all relative to LFD sedentary mice, data are presented as means ± SD and main effects are presented on graphs only if they are either close to or are significant. A two-way factorial ANOVA was used to examine main effects for diet and exercise as well their interaction. *Tukey* correction was used for multiple pairwise comparisons.

In contrast to the iWAT doublet banding pattern at 28 and 30 kDa (Figure 2F) when sclerostin was immunoblotted, eWAT only had 1 band at 30 kDa (there were 2/20 samples with detection of the 28 kDa band) (Figure 3F). Thus, only analysis of the 30 kDa band is presented. There was a main effect for diet (difference in predicted means of LFD and HFD = −114.9%, *p* = <0.0001), while there was no main effect for exercise (difference in predicted means from pre-to post-exercise = +17.7%, *p* = 0.2) and no interaction (*p* = 0.1) in the 30 kDa band of sclerostin within eWAT. The main effect for diet was a result of the 30 kDa monomer of sclerostin being nearly undetectable in eWAT of HFD fed mice (Figure 3A). It is important to note that there were detectable bands at 30 kDa in recombinant sclerostin when compared to eWAT samples (Supplementary Figure S2) and at sclerostin’s dimer predicted molecular weight of 56 kDa (Supplementary Figure S3).

Similar to iWAT, we examined components of Wnt signaling by assessing we assessed Wnt mediated inactivation phosphorylation of GSK and β-catenin accumulation. There were no main effects for either exercise (difference in predicted means from pre-to post-exercise = +5.2%, *p* = 0.8) or diet (difference in predicted means of LFD and HFD = +12.9%, *p* = 0.6) and no interaction (*p* = 0.3) for total GSK3β content within eWAT (Figure 3B). Likewise, there were no main effects for either exercise (difference in predicted means from pre-to post-exercise = −6.3%, *p* = 0.7) or diet (difference in predicted means of LFD and HFD = +17.8%, *p* = 0.2) and no interaction (*p* = 0.3) for serine9 phosphorylation of GSK3β within eWAT (Figure 3C). There were no significant main effects for either exercise (difference in predicted means from pre-to post-exercise = −0.1%, *p* = 0.9) or diet (difference in predicted means of LFD and HFD = −1.5%, *p* = 0.9) and no interaction (*p* = 0.07) for serine9 phosphorylation over total GSK3β within eWAT (Figure 3D). There were no main effects for either exercise (difference in predicted means from pre-to post-exercise = −2.2%, *p* = 0.5) or diet (difference in predicted means of LFD and HFD = +12.9%, *p* = 0.9) and no interaction (*p* = 0.7) was found for total β-catenin content within eWAT (Figure 3E). Figure 3F shows representative immunoblots for Wnt signaling related proteins in eWAT from each group.

## Discussion

In this study, we show new evidence that sclerostin is elevated in the circulation during recovery from acute exercise only in HFD induced obese and insulin resistant mice. When looking at the adipose tissue specific responses, we found that in iWAT, sclerostin decreased following exercise, independent of diet; however, eWAT sclerostin content was not responsive to exercise and was no longer detected after the HFD. Moreover, revealing downstream markers of the Wnt signaling pathway (GSK3β and β-catenin content) identified differential responses to HFD between iWAT and eWAT. Together, these results provide novel context to sclerostin’s endocrine role, which appears to be responsive to acute exercise (iWAT) and diet induced obesity/insulin resistance (eWAT) depending on the depot. We also present differences in molecular weights of sclerostin between WAT depots and diets, providing support for tissue specific and diet dependent regulation of sclerostin’s post-translational modifications.

Circulating sclerostin’s response to acute and chronic exercise has been extensively studied in humans, while there is no evidence in mice. In young adult humans (∼30 years of age), circulating sclerostin increases immediately following an acute bout of exercise, independent of mechanical loading in both male and female young adults (Kouvelioti et al., 2019). Older adults with osteoporosis also have an acute increase in sclerostin in response to walking/resistance exercise (Gombos et al., 2016). However, postmenopausal women without osteoporosis showed no acute sclerostin response to plyometric exercise but have higher levels of resting sclerostin compared to young adult women (Nelson et al., 2020). Our lab has also shown that normal weight healthy children have no response of circulating sclerostin to acute exercise, while those with obesity have an adult-like, transient post-exercise increase (Dekker et al., 2017; Kurgan et al., 2020). Taken together, there appears to be protection from an increase in circulating sclerostin in healthy children (likely a result of increased bone formation) and healthy older adults (inability to mobilize bone turnover), which is lost in individuals with perturbed metabolism or bone status. This is consistent with our present findings, where HFD mice had increased levels of sclerostin 2 h post-exercise, while LFD mice were unresponsive to acute exercise. There were no differences in sclerostin at rest in HFD mice compared to LFD mice, which is in contrast to previous studies that have shown that a HFD increases bone sclerostin expression (Baek et al., 2014) leading to increased circulating sclerostin (Kim et al., 2017). Since we used the same strain and sex of mice, these differences may be due to age or the length of the HFD, as these studies used 8 weeks of HFD with mice ∼16 weeks of age, while we used 10 weeks of a HFD with same strain mice 18 weeks of age (24 vs. 28 weeks of age at analysis).

The identification of sclerostin’s endocrine role in regulating fat mass (Kim et al., 2017; Kim et al., 2019; Kim et al., 2021) and HFD mice circulating sclerostin increasing following acute exercise led us to examine the influence of fat mass expansion and perturbed insulin sensitivity (HFD vs. LFD fed mice) on the response/mobilization of sclerostin within adipose tissue at rest and in response to acute exercise. In contrast to peripheral sclerostin, there appears to be a reduction in monomeric sclerostin content (both 28 and 30 kDa bands) within iWAT following acute exercise independent of diet. While there was no significant effect of acute exercise on iWAT GSK3β serine9 phosphorylation, there was a trend (*p* = 0.07) for an increase in β-catenin content, which is expected with less sclerostin and increased Wnt signalling. The timing of sampling is likely why we only observed a trend (*p* = 0.07) for an increase in β-catenin content following acute exercise. This response is likely temporal, as our study examined the response 2 h post-exercise and previous studies have shown Wnt/β-catenin signaling within adipose tissue increases immediately following exercise (Shen et al., 2016). While the mechanism of sclerostin “mobilization” into tissue or “uptake” into cells is unclear, there is evidence that sclerostin may be mobilized as a dimer (Hernandez et al., 2014) or shuttled to peripheral cells by extracellular vesicles (Morrell et al., 2018). Moreover, RNA profiling does show very low expression of Sost sclerostin in subcutaneous adipose tissue of *Mus musculus* (Gene ID: 74499 in NIH GEO Profiles). However, it is important to note that when there is conditional knockout of sclerostin within osteoblasts there is nearly an 80% reduction in circulating sclerostin while adipocyte conditional knockout models have no influence on circulating sclerostin (Kim et al., 2019; Kim et al., 2021). Therefore, it is likely that the relatively large changes in adipose tissue sclerostin content observed in this study are reflective of changes in bone expression/secretion.

It is interesting to note that iWAT total GSK3β and β-catenin content were both higher in HFD mice compared to LFD mice. With its role in the Wnt signalling pathway, an increase in GSK3β would theoretically lead to reduced levels of β-catenin (Liu et al., 2005). However, our data suggests otherwise, where HFD enhanced Wnt signalling independent of changes in total GSK3β. This is likely a compensatory response to changes in the regulation of Wnt signaling with HFD feeding aimed at enhancing lipolysis in the face of excessive adiposity. This compensation is likely regulated by other components of Wnt signaling (e.g., Wnt’s co-receptors LRP 5/6). In addition, it has been shown that HFD feeding in adipocyte specific β-catenin knockout mice can compensate for this inhibited Wnt signaling by increasing β-catenin expression by stromal-vascular cells within adipose tissue, which can be used to rescue Wnt signaling within adipocytes (Bagchi et al., 2020). Furthermore, an increase in GSK3β content is not entirely surprising given its role in adipogenesis and metabolism where it has been shown that GSK3β can inhibit expression of the thermogenic gene program (Markussen et al., 2018), and increase inflammation (Wang et al., 2018) and adipogenesis (Benjamin et al., 1994; Ross et al., 1999; Zaragosi et al., 2008). We propose that GSK3β increases content in iWAT in response to HFD feeding to increase adipogenesis, and acute exercise can counteract this by increasing Wnt signaling (trend for increased β-catenin content), which may be regulated in part by reduced sclerostin.

Since sclerostin knockout models are protected against an obesogenic diet in both subcutaneous and visceral fat pads by inhibiting adipogenesis and increasing insulin sensitivity and fat oxidation (Kim et al., 2017), we hypothesized that a HFD would increase sclerostin content in both WAT depots due to reduced insulin sensitivity. In contrast, we found monomeric sclerostin content was unresponsive in iWAT and undetectable in eWAT of HFD mice compared to LFD fed mice at rest. However, this does not mean sclerostin was not present in eWAT, as there was a strong band at ∼56 kDa (Supplementary Figure S3), representing a sclerostin dimer (Hernandez et al., 2014). We suspect that there is either a push to preferentially form dimers and reduce monomeric sclerostin content or there is a reduction in the mobilization of monomeric sclerostin to visceral WAT while subcutaneous WAT is not impacted. There are no crystallized structure images on protein data bank (Berman et al., 2000) of the sclerostin dimer, which prevents speculation on the influence of changes in glycosylation or dimerization on protein function. Imaging sclerostin’s dimer structure or predicting its structure through computational methods (Brini et al., 2019) would provide insight into the influence dimerization would have on sclerostin’s ability to interact with its LRP6 epitope (Kim et al., 2020) and enable comparison of dimeric sclerostin’s inhibition of Wnt function compared to monomeric sclerostin. It is important to note that eWAT sclerostin and components of Wnt/β-catenin signaling were unresponsive to acute exercise in this study, suggesting sclerostin may not influence eWAT’s long term adaptations to diet or exercise, which may be due to alterations in its sensitivity to sclerostin action (e.g., receptor content—LRP4 and 6 (Kim et al., 2019; Kim et al., 2020). The importance of these changes to sclerostin’s content and post-translational modifications with exercise and HFD feeding are critical in furthering our understanding of its role in regulating bone (Poole et al., 2005), adipose tissue (Kim et al., 2017), and skeletal muscle (Magarò et al., 2021) growth and metabolism, which we plan to directly examine in future studies.

There are several limitations to the present study design, that limits our analysis and interpretation to correlations in the response of sclerostin and Wnt signaling. Thus, our conclusions lack mechanistic insight or causative effects of changes in sclerostin content and Wnt signaling within adipose tissue and their influence on growth and metabolism. Rather, we present responses in proteins with known functions in these tissues providing preliminary evidence for their role in adaptations to diet and exercise and rationale for further examination. Additionally, assessment of more post-exercise time points, particularly immediately post-exercise, is needed to fully understand the temporal behavior of sclerostin and Wnt signaling acutely post-exercise. WAT is also comprised of mature adipocytes, preadipocytes, endothelial, stromal, and immune cells, thus it is important to consider the changes we are observing may be a result of changes occurring in any of these cell types (Bagchi et al., 2020). Cell specific responses to high fat feeding and acute exercise is important to elucidate which cell types are contributing to these responses. Importantly, since our data include whole tissue lysate, we cannot answer the question whether sclerostin is bound to receptors on adipocytes or whether is expressed in other cell types that are present in adipose tissue. Another limitation is that the study design lacks negative control tissues such as protein extracted from a sclerostin knock out mouse. We also only report responses in male mice, which neglects sex specific differences. Lastly, an important caveat is that GSK3β activity is not solely regulated by serine9 phosphorylation status or content and Wnt signaling does not require its phosphorylation to inhibit its kinase activity for β-catenin as its substrate (Piao et al., 2008). Although phosphorylation of GSK3β can occur in tandem, increased β-catenin content is an indicator of canonical Wnt signal transduction.

Given sclerostin’s association with fat oxidation and insulin sensitivity, future studies are needed that examine sclerostin’s response to changes in adipose tissue lipolysis (e.g., induced by catecholamines or models of inhibited WAT lipolysis) or insulin stimulated glucose uptake. These studies will provide mechanistic insight into sclerostin’s mobilization with exercise and the differential response between fat pads. Assessment of the importance of sclerostin reduction within iWAT with multiple bouts of acute exercise (i.e., exercise training) is also needed to understand its role in regulating adipose tissue mass and adipocyte cell size. Lastly, assessing eWAT and iWAT *ex vivo* explants differences in Wnt signaling activation to varying doses of Wnt ligands (e.g., Wnt 3a) and sclerostin will allow for further understanding of depot specific sensitivity to Wnt signaling and sclerostin action (e.g., receptor content).

## Conclusion

Herein we have characterized the monomeric sclerostin response to acute exercise as well as the influence of HFD feeding on this response. While HFD and LFD mice have no difference in resting levels, HFD augments circulating sclerostin response to acute exercise, which remains unresponsive in LFD mice. Within visceral eWAT, monomeric sclerostin content is abolished and is unchanged in iWAT in HFD mice compared to LFD mice, while acute exercise does not influence eWAT sclerostin and leads to a reduction in iWAT independent of diet. Thus, sclerostin is responsive to acute exercise (iWAT) and fat mass expansion (eWAT), conferring a contribution of this bone derived endocrine factor in regulating WAT growth and metabolism that requires further examination.

## Data Availability

The datasets for this study can be found in the Brock University Library Repository (http://hdl.handle.net/10464/16731).
