# Supplementary material for: Characterization of sclerostin’s response within white adipose tissue to an obesogenic diet at rest and in response to acute exercise in male mice
Source: Front Physiol. 2023 Jan 4;13:1061715. doi: 10.3389/fphys.2022.1061715 (PMC9846496; doi:10.3389/fphys.2022.1061715)
Supplement: Supplementary file 1 [file DataSheet2.PDF]

Supplementary Table 1. List of materials used.

Material	Catalogue number	Source
C57BL/6 mice		The Jackson Laboratory (Bar Harbor, Main, USA)
Standard chow (Teklad global 14% protein rodent maintenance diet)		Harlan Tekland (Mississauga, Ontario, Canada
High-fat diet (60% kcal fat)	D12492	Research Diets Inc. (New Brunswick, New Jersey, USA)
Low-fat diet (10% kcal fat)	D12450B	Research Diets Inc. (New Brunswick, New Jersey, USA)
Serum sclerostin Quantikine enzyme-linked immunoassay kit	MSST00	R&D Systems (Minneapolis, Minnesota, USA)
iWAT and eWAT depot homogenization	FastPrep®	MP Biomedicals (Santa Ana, California, USA)
NP40 Cell Lysis Buffer	FNN0021	Life Technologies (Carlsbad, California, USA)
Phenylmethylsulfonyl fluoride and protease inhibitor cocktail	7626 and P8340	Sigma-Aldrich (St.Louis, Missouri, USA)
Bicinchoninic acid assay	B9643	Sigma-Aldrich (St. Louis, Missouri, USA)
Copper (II) sulfate pentahydrate	BDH9312	VWR (Radnor, Pennsylvania, USA)
Laemmli buffer	1610747	Bio-Rad (Hercules, California, USA)
TGX fast cast gels	1610173	Bio-Rad (Hercules, California, USA)
Polyvinylidene difluoride membrane	Trans-Blot® Turbo™ Transfer System	Bio-Rad (Hercules, California, USA)
Horseradish peroxidase	Anti-rabbit: HAF008<br>anti-goat: CAF109	R&D Systems (Minneapolis, Minnesota, USA)
Clarity™ Western chemiluminescent substrate	170-5061	Bio-Rad (Hercules, California, USA)
SuperSignal™ West Femto maximum sensitivity chemiluminescent substrate	34095	ThermoFisher Scientific (Waltham, Massachusetts, USA)
ChemiDoc™ Imaging System		Bio-Rad (Hercules, California, USA)
ImageLab Software		Bio-Rad (Hercules, California, USA)
Vinculin	ab129002	Abcam (Toronto, Ontario, Canada)
Total GSK3β antibodies	12456S	Cell Signalling (Danvers, Massachusetts, USA)
Serine 9 phospho-GSK3β antibodies	9336S	Cell Signalling (Danvers, Massachusetts, USA)
Total β-catenin antibodies	8480S	Cell Signalling (Danvers, Massachusetts, USA)
Sclerostin antibody	MAB1406	R&D Systems (Minneapolis, Minnesota, USA)
Femur homogenates and recombinant sclerostin	1406	R&D Systems (Minneapolis, Minnesota, USA)
