# Supplementary material for: Characterization of sclerostin’s response within white adipose tissue to an obesogenic diet at rest and in response to acute exercise in male mice
Source: Front Physiol. 2023 Jan 4;13:1061715. doi: 10.3389/fphys.2022.1061715 (PMC9846496; doi:10.3389/fphys.2022.1061715)
Supplement: Supplementary file 2 [file DataSheet1.PDF]

Supplemental Figure 1

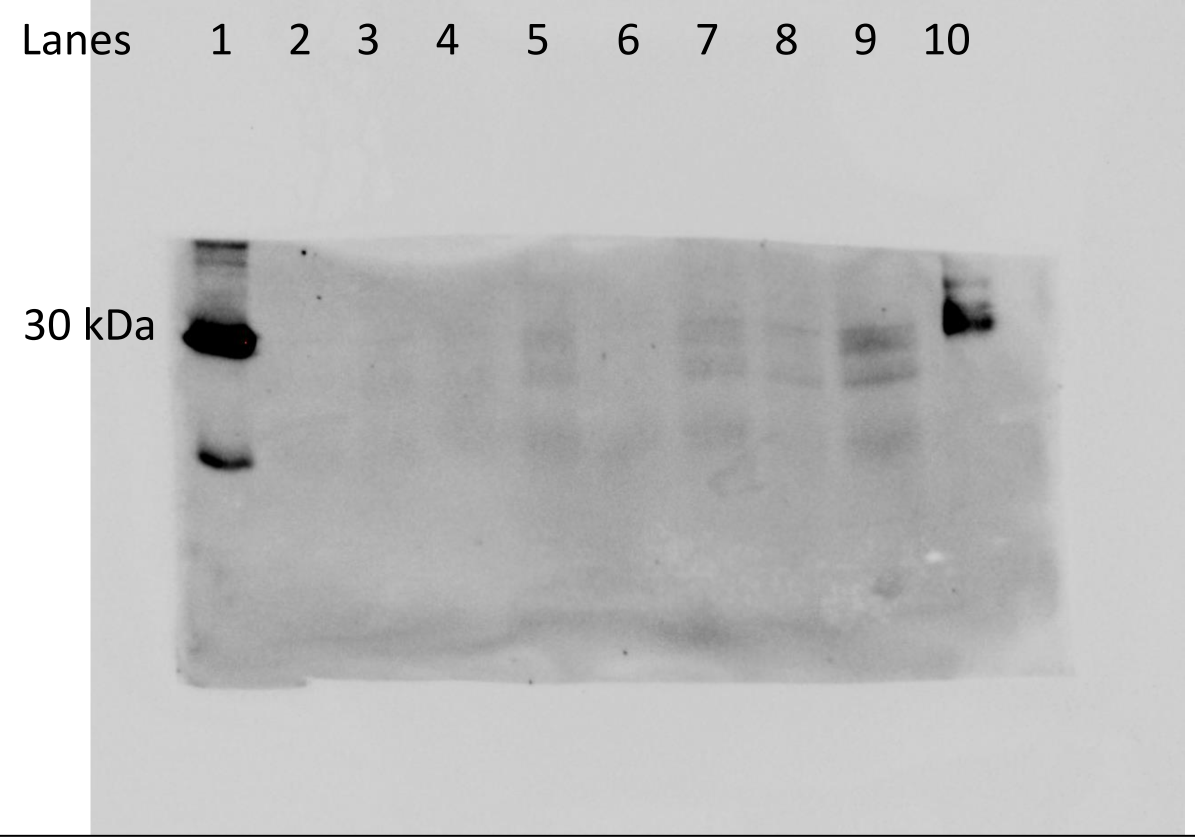

Tissue: Femur

Conditions: 12.5% gels, electrophoresis at 150V for 5 min then 90V for 1h 20min, transfer with trans blot turbo bio rad std protocol for 30 min, blocked in 5% non-fat milk for 1h, primary overnight at 4C, secondary for 1.5h, substrate = super signal west femto, imaged with biorad imager for chemi – setting = auto optimal

Protein of interest: Sclerostin – primary: R&D (AF1589); mouse, polyclonal goat IgG reconstituted to 1 ug/ul; dilution of 1:500 (10ul in 5 ml in 5% non-fat milk = 2 ug/ml). Secondary: R&D (AF109); goat, polyclonal Donkey IgG HRP-conjugated antibody reconstituted to 1:2000 dilution in 5% non-fat milk

Samples: Lanes; 1 = MW, 2 = 1LFD, 3 = 2LFD, 4 = 1HFD, 5 = 2HFD, 6 = 1LFD, 7 = 2LFD, 8 = 1HFD, 9 = 2HFD, 10 = recombinant sclerostin (+C)

Protein load: Lane 1 = MW marker (4 ul), lanes 2-5 = 7 ug, lanes 6-9 = 15 ug, and lane 10 = 15 ul

Notes:

- Membrane is cut at 50 kDa to focus exposure on monomeric sclerostin.

Supplemental Figure 2

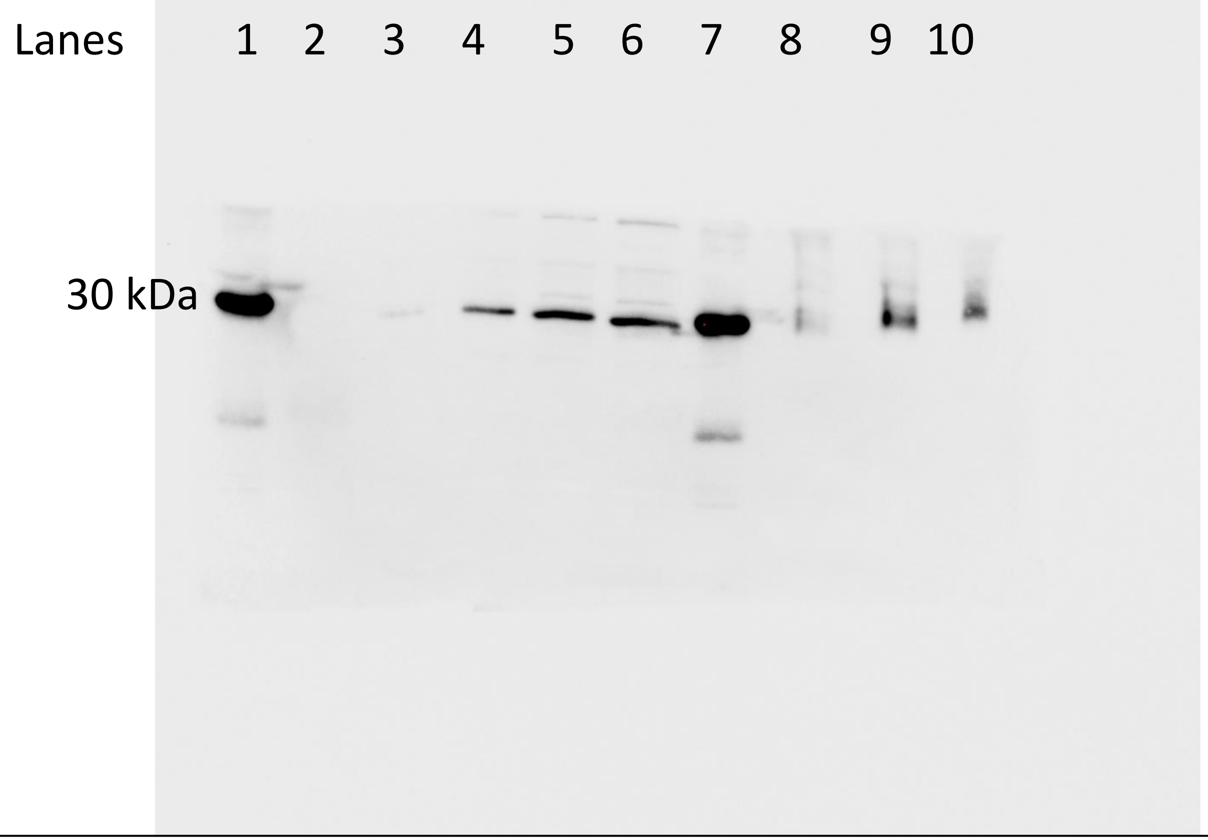

Tissue: eWAT, control

Conditions: 12.5% gels, electrophoresis at 150V for 5 min then 90V for 1h 20min, transfer with trans blot turbo bio rad std protocol for 30 min, blocked in 5% non-fat milk for 1h, primary overnight at 4C, secondary for 1.5h, substrate = super signal west femto, imaged with biorad imager for chemi – setting = auto optimal exposure

Protein of interest: Sclerostin – primary: R&D (AF1589); mouse, polyclonal goat IgG reconstituted to 1 ug/ul; dilution of 1:500 (10ul in 5 ml in 5% non-fat milk = 2 ug/ml). Secondary: R&D (AF109); goat, polyclonal Donkey IgG HRP-conjugated antibody reconstituted to 1:2000 dilution in 5% non-fat milk

Samples: Lanes; 1 = MW (4 ul), 2 = LFD femur, 3-6 = LFD eWAT, lane 7 = MW (2 ul), lane 8-10 = recombinant sclerostin (C)

Protein load: Lane 1 = MW marker (4 ul), lane 2 = 5 ug, lane 3 = 5 ug, lane 4 = 10 ug, lane 5 = 20 ug, lane 6 = 30 ug, lane 7 = MW Marker (4 ul), lane 8 = 10 ul +control, lane 9 = 20 ul +control, lane 10 = 30 ul +Control (+Control = recombinant sclerostin)

Notes:

- Femur sample band is being crowded by ladder over exposure.
- For eWAT load 20 ug for clean bands. Membrane is cut at ~50 kDa to focus exposure on monomeric sclerostin

Supplemental Figure 3

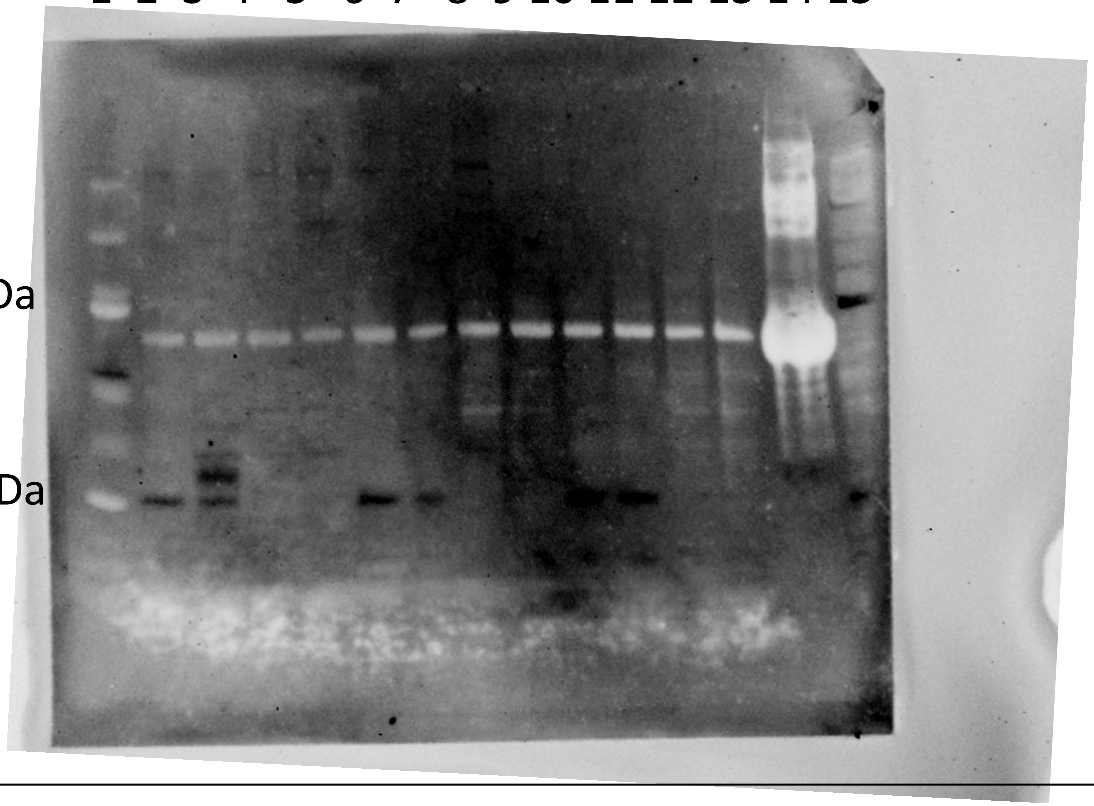

Tissue: eWAT

Conditions: 10% TGX fast cast gels, electrophoresis at 250V for 26 min, transfer with trans blot turbo bio rad turbo mini gels at 7 min, blocked in 5% non-fat milk for 1h, primary for 1h at RT, secondary for 1h, substrate = super signal west femto, imaged with biorad imager for chemi – setting = auto optimal exposure

Protein of interest: Sclerostin – primary: R&D (AF1589); mouse, polyclonal goat IgG reconstituted to 1 ug/ul; dilution of 1:1000. Donkey IgG HRP-conjugated antibody reconstituted to 1:5000 dilution in 5% non-fat milk

Samples: Lanes; 1 = MW, 2 = LS6, 3 = LE6, 4 = HS6, 5 = HE6, 6 = LS7, 7 = LE7, 8 = HS7, 9 = HE7, 10 = LS8, 11 = LE8, 12 = HS8, 13 = HE8, 14 = recombinant sclerostin, 15 = brain

Protein load: Lane 1 = MW marker (2 ul), lanes 2-13 = 9 ug, lane 14 = 15 ul, LANE 15 = 10 ug

Notes:

- There is a prominent dimer, even in recombinant sclerostin. Haloing indicates too much substrate/exposure.
